# Methods to accelerate PROTAC drug discovery

**DOI:** 10.1042/BCJ20243018

**Published:** 2025-06-25

**Authors:** Jeyan Osman, Philip E. Thompson, Manuela Jörg, Martin J. Scanlon

**Affiliations:** 1 Medicinal Chemistry, Monash Institute of Pharmaceutical Sciences, Monash University, Parkville, VIC, Australia; 2 ARC Training Centre for Fragment Based Design, Monash Institute of Pharmaceutical Sciences, Monash University, Parkville, VIC, Australia; 3 Chemistry-School of Natural and Environmental Sciences, Newcastle University, Newcastle Upon Tyne, U.K.

**Keywords:** click chemistry, direct-to-biology, DNA-encoded library, proteolysis-targeting chimeras, solid-phase synthesis

## Abstract

Proteolysis-targeting chimeras (PROTACs) represent a novel and promising modality for probing biological systems, elucidating pharmacological mechanisms, and identifying potential therapeutic leads. The field has made significant strides, as demonstrated by the growing number of PROTACs advancing to clinical trials. Despite this progress, the development of PROTACs faces significant challenges, which is partially due to the heterobivalent nature of this class of molecules. PROTACs must simultaneously bind to a protein of interest and an E3 ubiquitin ligase. This means PROTACs are significantly larger and more complex than conventional small molecules. This complexity impacts their design and synthesis, requiring strategic approaches to create libraries of PROTACs with various combinations of target ligands, linkers, and E3 ligase-recruiting elements. To fully realise the potential of this innovative technology, there is a need for novel approaches to accelerate the development of PROTACs. This review focuses on three critical areas to accelerate PROTAC development: appropriate target selection, modular chemical synthesis, and high-throughput biological evaluation. By appropriate selection of target proteins for degradation, optimizing synthesis methods to handle the complexity of PROTAC molecules, and employing robust high-throughput biological assays to evaluate PROTAC activity, researchers in academia and industry have streamlined the development and increased the success rate of PROTAC-based discovery programmes.

## Introduction

**Figure 1:**
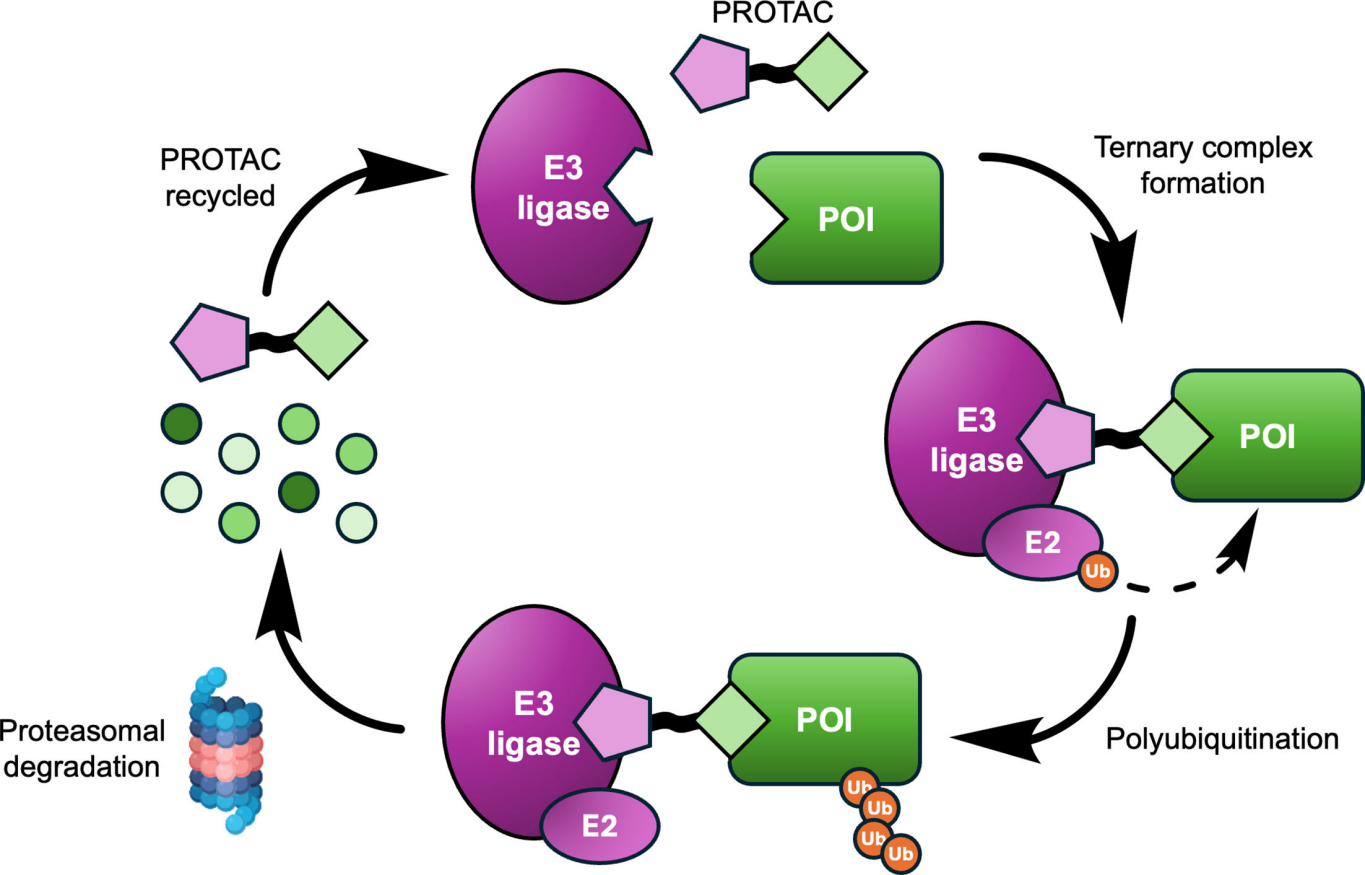
Schematic representation of PROTAC mechanism of action. The PROTAC engages both the E3 ligase and the POI in a ternary complex. This allows an E2 conjugating enzyme to polyubiquitinate the POI, which labels the POI for proteasomal degradation. The PROTAC is released and recycled.

Targeted protein degradation (TPD) has become established over the past decade as an exciting new modality with applications in chemical biology and drug discovery [1–5]. Although there are now several different approaches that can achieve TPD, one of the most prevalent is the use of proteolysis-targeting chimeras (PROTACs), which is the focus of the current review. PROTACs are heterobifunctional molecules that comprise three distinct elements [6,7]: a ligand that binds to a protein of interest (POI), a ligand that binds to an E3 ubiquitin ligase, which is referred to as an E3 recruiting element (E3RE), and a linker that connects the two. E3 ligases form part of the ubiquitin–proteasome system, which is part of the cell’s natural protein degradation machinery. PROTACs act as chemical inducers of proximity that promote the formation of a ternary complex between the PROTAC, POI, and E3 ligase. By inducing proximity to the E3 ligase, this enables the transfer of ubiquitin onto the POI, which labels the POI for degradation by the proteasome (Figure 1). Afterwards, the PROTAC is released, allowing it to be recycled so that it can act catalytically [6,8,9].

Although there are >600 E3 ligases found in the human body [10], the vast majority of PROTACs that have been reported target only two E3 ligases, i.e. von Hippel-Lindau E3 ligase (VHL) and cereblon (CRBN) [11]. The subsequent sections describing methods to accelerate synthesis and testing of PROTACs reflect this. Nonetheless, there are an increasing number of E3RE ligands that target a broader range of E3 ligases [12]. Although these E3REs are not explicitly discussed in the current review, many of the approaches described for PROTAC discovery are modular and, therefore, can be effectively applied to a range of POI ligands, linkers, and E3REs.

PROTACs offer several advantages over conventional chemical inhibitors—both as chemical probes of protein function and as therapeutic lead compounds. Unlike conventional small-molecule inhibitors that block a protein’s activity, PROTACs result in protein degradation and, thereby, inhibit not only the activity but also all other functions of the protein, e.g. in scaffolding of complexes. In this way, they mimic the effects of genetic deletion of a protein. However, they can work much more rapidly, which reduces the risk of the cell compensating by altering the expression of related genes. This mechanism of action has two important consequences. First, PROTACs exhibit ‘event-driven pharmacology’, since each PROTAC can degrade multiple copies of the POI. Therefore, the requirement to achieve stoichiometric binding to the POI is removed, and the PROTAC effectively exhibits a catalytic mechanism. This removes the requirement to achieve threshold occupancy to elicit the desired response. These features lead to the second consequence—that PROTACs can be used to target proteins that were previously considered to be ‘undruggable’—such as those without deep pockets suited to the binding of small molecules, because efficacy can be achieved even with low occupancy of the POI target [13–15]. For example, Testa et al. developed PROTACs targeting Bromodomain and Extra Terminal (BET) proteins by VHL recruitment. One of these PROTACs formed a binary complex with VHL that had an affinity of 603 nM, yet it was able to degrade bromodomain-containing protein 4 (Brd4) with a DC_50_ between 10 and 30 nM [16]. Similarly, a p38α-targeting PROTAC was able to degrade with a DC_50_ value of 210 nM while having a binary affinity of only 11 µM [17]. Moreover, the POI ligand of the PROTAC does not need to bind to the active site of the protein in order to have a therapeutic effect. The POI binder must simply promote the formation of a ternary complex where the E3 ligase has convenient access to a proximal lysine residue for effective ubiquitination. A compelling example is the epidermal growth factor receptor (EGFR) L858R-selective degrader CFT8919, which binds to an allosteric site rather than the ATP-binding pocket. This non-catalytic binding allows it to selectively degrade the mutant form of EGFR, including drug-resistant variants, without affecting the wildtype protein—demonstrating how PROTACs can exploit unique structural features for precision targeting beyond traditional inhibition [18].

**Figure 2:**
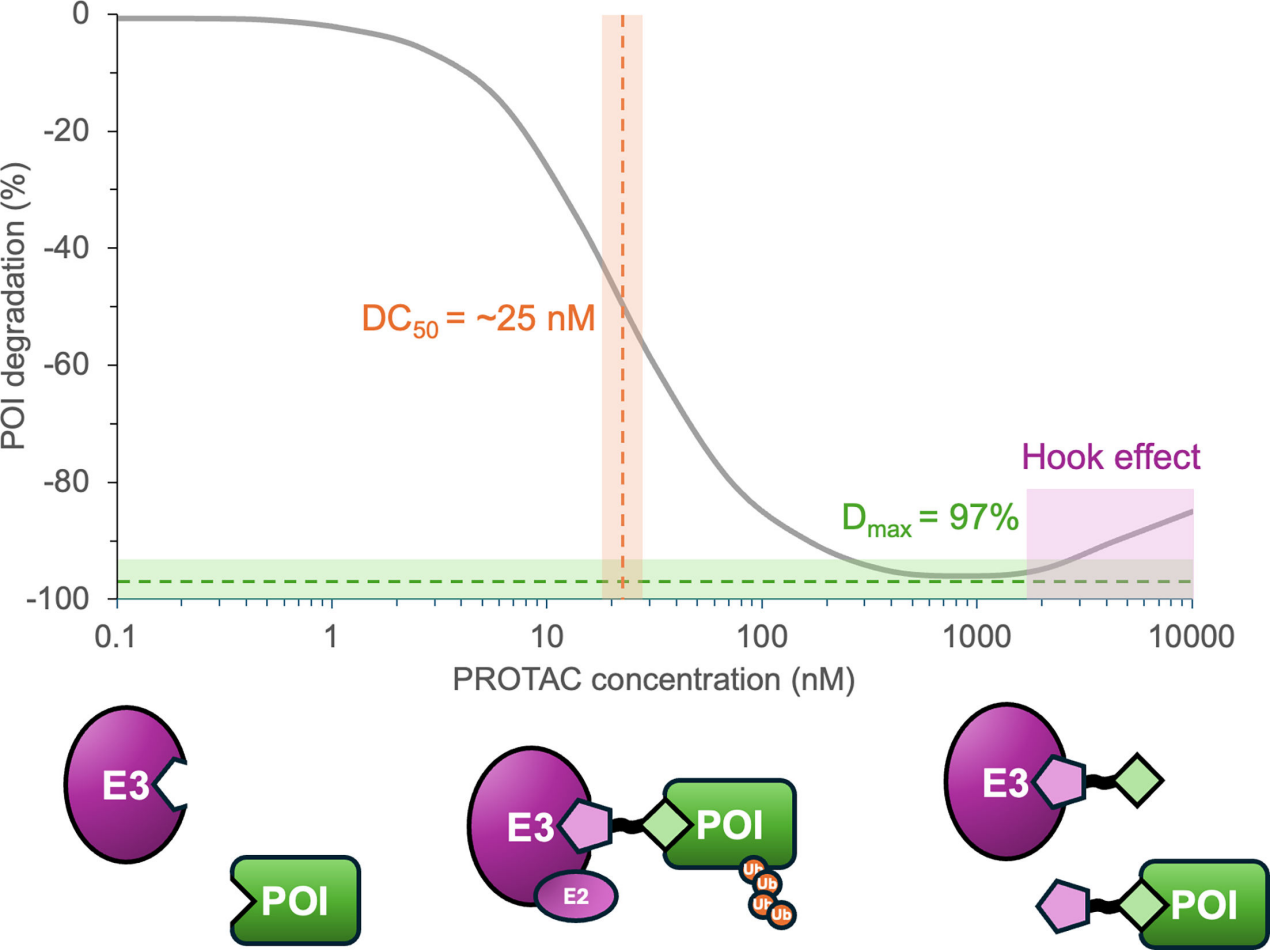
Simulated characterisation of PROTAC efficacy. The curve shows POI degradation as a function of PROTAC concentration. DC_50_ (orange) and D_max_ (green) values are shown with respective relationship to the corresponding datapoints on the curve. The Hook effect is highlighted in purple, where, at higher PROTAC concentrations, the binary complexes of POI–PROTAC and PROTAC–E3 compete with ternary complex formation.

For these reasons, PROTAC efficacy is not measured by target occupancy but by the ability to elicit degradation of the target (Figure 2). The cellular concentration of the POI can be measured in several different ways, ranging from measurement by western blotting [19–21] to more high-throughput and/or real-time luminescence-based assays [22–25]. These allow measurement of D_max_, which is the maximal degradation, and other parameters such as the DC_50_, which is the PROTAC concentration at which 50% of the D_max_ is achieved.

In practice, the efficacy of a PROTAC is dictated by a complex interplay of factors, including the affinity and kinetics of binding to the E3 ligase and to the POI in the two binary complexes, the presence of any binding co-operativity in the ternary complex, as well as the rates of ubiquitination and proteasomal degradation [26]. The need to form a stable ternary complex to elicit POI degradation can lead to the observation of ‘Hook effects,’ where the degradation efficiency decreases at higher concentrations of PROTAC (Figure 2). This paradoxical effect is due to the preferential formation of binary complexes, where the PROTAC binds to either the POI or E3 ligase alone, rather than forming a ternary complex. The requirement for ternary complex formation is a key feature that enables PROTACs to achieve selective degradation even when starting from pan-selective ligands. This is because the formation of a productive ternary complex depends not only on the binary affinities of the PROTAC for the E3 ligase and the POI but also on the co-operative interactions between all three components. For example, MZ1, a PROTAC targeting BRD4, was derived from the pan-BET inhibitor JQ1, yet it selectively degrades BRD4 over other BET family members due to favourable ternary complex formation with VHL and BRD4 [9,27]. This selectivity arises from the unique surface complementarity and co-operative binding in the ternary complex.

Developing PROTACs presents significant challenges, since it requires the identification of two suitable ligands, which must be linked appropriately [28]. The linker must be precisely engineered to maintain spatial orientation and flexibility of the two ligands to ensure effective simultaneous engagement with both the POI and E3 ligase. Furthermore, achieving adequate bioavailability, stability, and minimising off-target effects requires precise molecular design and thorough testing. Developing more systematic approaches to generate PROTACs is vital for the development of more effective tools to probe biological systems, to better understand the molecular mechanisms of pharmacological control and ultimately to accelerate the discovery of compounds that have potential as therapeutic lead molecules [29]. The current review covers three specific areas where methods have been reported to accelerate the process of developing PROTACs. These cover target selection through to assessment of activity *in cellulo*. First, we provide an overview of methods to assess the suitability of a target POI for PROTAC development. Secondly, we review different methods that have been developed to accelerate the chemical synthesis of potential PROTAC molecules. Finally, we describe some of the ‘direct-to-biology’ (D2B) approaches that enable high-throughput evaluation of the ability of compounds to degrade the POI in a cellular context and how these have been adapted to accelerate PROTAC development.

There are many additional challenges involved in taking a PROTAC that degrades a POI in a cellular assay through preclinical and clinical development as a potential therapeutic [30,31]. However, these are beyond the scope of the current review.

### Section 1 – Selection & validation of target proteins of interest

Careful target selection is fundamental to the successful development of all drugs, including PROTACs, as it influences their therapeutic efficacy, safety, and overall clinical potential. Understanding the protein’s role in disease pathology, its interactome and potential resistance mechanisms can inform the selection of suitable targets and the development of more effective PROTACs. For example, in cancer therapy, targeting a mutant oncoprotein that is present in malignant cancer cell lines (such as KRAS^G12C^ or BRAF^V600E^) can maximise therapeutic efficacy while minimising off-target effects [32–35]. However, in many cases, the target POI is expressed in both healthy and diseased cells. To gauge the tractability of generating a PROTAC for a specific POI, several experimental and computational methods have been developed to evaluate a target protein’s degradability and the resulting biological outcomes. In general, these can be used to assess a POI prior to embarking on the task of developing a specific PROTAC against that target.

#### Tag-targeted protein degraders

**Figure 3:**
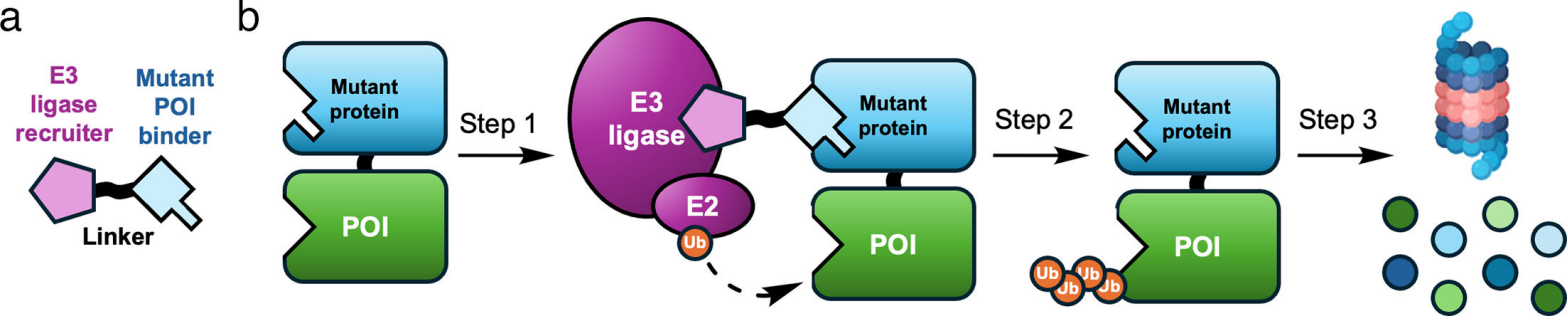
Schematic of a tTPD system. (**a**) Representative structure of a tTPD. (**b**) Steps in the mechanism of a tTPD system. The respective proteins are labelled and ubiquitin is shown as orange spheres.

Fusion-protein tag-based systems can simulate degradation and its consequences, helping to pre-emptively assess the feasibility of PROTAC-mediated TPD. These ‘tag-TPD’ (tTPD) systems generally consist of a ‘mutant protein’ (MP) for which a TPD-inducing ligand is already available (Figure 3). Numerous tTPD systems, such as such as dTAGs [36], BromoTAGs [37], and HaloTAGs [38–40], (targeting FKBP12^F36V^, Brd4BD1^L94^, Brd4BD2^L387^, and bacterial haloalkane dehalogenase, respectively) have been reported in the literature and/or are commercially available to study protein function in cells. These systems enable efficient assessment of protein degradability without requiring POI-specific ligands. tTPDs employ heterobivalent tool compounds, which comprise a binder of a MP and a binder of an E3 ligase (Figure 3). By expressing the MP as a fusion with a specific POI, these tTPD technologies can, in principle, degrade any POI, without the requirement of generating a PROTAC that is specific for the POI. These tTPDs largely work via the same mechanism; the heterobivalent tTPDs can bring an engineered MP-POI fusion into proximity to an E3 ligase (step 1, Figure 3), thus facilitating ubiquitination of the MP-POI complex (step 2, Figure 3), and subsequent protein degradation via the proteasome (step 3, Figure 3). These tTPD tools have certain benefits over traditional genetic knockdown or knockout technologies, as they are directly targeting proteins (instead of acting at the genome level) and providing rapid, reversible, and temporal control of protein abundance [29,41].

The tTPD technologies provide a system to address the feasibility of degrading a specific POI and potentially investigating the consequences of degradation. Assessing the consequences of POI degradation is important, because in contrast with most small-molecule or peptide inhibitors that impede POI function by occupying an active site, PROTACs result in the loss of the protein from the cell. Therefore, PROTACs can inhibit not only the specific activity of a POI but also any other functions that the POI performs, e.g. scaffolding. For example, PROTACs targeting receptor tyrosine kinases (RTKs) have been demonstrated to be superior to traditional inhibitors by providing more effective and sustained suppression of cell proliferation. This is attributed in part to the fact that PROTACs can target kinase-independent functions of RTKs, potentially leading to more comprehensive treatment outcomes [42].

#### Bioinformatics and computational approaches

Bioinformatic and computational methods are increasingly important in target selection, as they allow for the rapid analysis of vast amounts of biological data. These methods can predict potential targets that are suitable for degradation based on protein structure, function, and interaction networks. Computational tools can help identify both the proteins that are crucial for disease progression and those likely to be degraded effectively by PROTACs. One example is PrePROTAC—a machine learning model designed to predict proteins that can be degraded by PROTACs [43]. Utilising its machine learning approach, PrePROTAC achieved high accuracy in identifying potential targets for the E3 ligase CRBN. Additionally, the study employed an embedding method termed ‘SHapley Additive exPlanations’ (eSHAP) to pinpoint key residues in protein structures that influence PROTAC activity. The research identified over 600 novel proteins that are potentially degradable by CRBN. In addition, three new potential POIs for PROTAC development in the treatment of Alzheimer’s disease were proposed. PrePROTAC focused on identifying new POIs that are degradable by CBRN. An alternative approach described by Liu et al. attempted to identify E3 ligases that were suited to the degradation of specific POIs. This involved systematic characterisation of hundreds of E3 ligases according to their ligandability, expression patterns, and predicted interaction networks. This analysis identified 76 E3 ligases as potential PROTAC-interacting candidates [12]. These are provided in a database (https://hanlaboratory.com/E3Atlas/), which describes the E3 ligases and characterises them by confidence score. This provides a resource to identify new potential E3 ligases that may be suitable for inducing TPD activity against specific POIs.

#### Proteomics

Proteomic profiling is an effective method to reveal all the proteins that are affected by PROTAC-mediated knockout of a specific POI. Mass spectrometry (MS)-based proteomics is a fast, sensitive, and quantitative way of measuring the abundance of thousands of protein species from biological samples in one experiment [44]. In the context of target selection, novel targets can be elucidated by deciphering which proteins are significantly altered in the disease state versus the healthy state, as exemplified by Sathe et al.’s work on Alzheimer’s disease [45]. Additionally, Sathe et al. published a review detailing proteomic approaches for TPD, which suggested that matching subcellular POI distribution to specific degradation compartments can enhance degradation. The subcellular distribution can be determined by separating organelles and analysing their protein content by MS-based proteomics [46,47]. There are also established chemoproteomic databases where potential POI targets have been reported, which also highlight examples where small-molecule binders have been described. For example, Donovan et al. reported a chemo-proteomics-based approach to identify a group of degradable protein kinases and kinase-like proteins. This was achieved by selecting small molecules that have been reported to bind a large proportion of the kinome. These compounds were attached via a diverse set of linkers to an E3 ligand that bound to either VHL or CRBN. The resulting PROTACs were tested for cellular permeability and deployed in PROTAC-induced degradation experiments across the human kinome to characterise the kinase degradation profile for each compound. This analysis generated a large library of protein kinase-targeting degraders [48]. The dataset provided a resource to explore characteristics required for efficient degradation. For example, the effect of target residence time on degradation was assessed [49]. This employed a series of PROTACs where the dissociation rate from the kinase POI was modulated by employing non-covalent, reversible covalent, and irreversible covalent warheads on a pan-kinase binding compound. This analysis revealed a complex interplay of the kinetics of binding and ubiquitination dictated the rate of degradation for different kinase targets, since no single parameter (e.g. affinity, co-operativity, and residence time) was sufficient to describe the rate of effective degradation.

#### PROTAC target scope

While PROTACs offer the prospect of targeting previously undruggable receptors, such as transcription factors, scaffold proteins, and regulatory enzymes, their development presents unique challenges. PROTACs must be able to form stable ternary complexes that induce proximity between the target POI and an E3 ligase [9]. Therefore, the POI and E3 ligase need to be in the same cellular compartment for ubiquitination and consequent degradation to occur. This can make it challenging to develop PROTACs against certain proteins, such as membrane proteins. For instance, G protein-coupled receptors (GPCRs) are a class of membrane-bound receptors that regulate a wide range of physiological processes and have proven to be tractable drug targets in the treatment of many diseases. Even though about a third of current therapies target GPCRs, the development of PROTACs for GPCRs is extremely challenging. This is due in part to the requirement that the PROTAC must interact with both the membrane-bound GPCR and an intracellular E3 ligase. However, there are alternative degradation approaches, including lysosome-targeting chimeras (LYTACs) and anti-body based PROTACs, which represent new archetypes that can degrade GPCRs by accessing lysosomal degradation pathways or recruiting membrane-bound E3-ligases, respectively [50,51]. These are beyond the scope of the current review and not discussed further. There has been some success with PROTAC-medicated degradation of integral membrane proteins; however, the mechanism of PROTACs makes them better suited for targeting intracellular proteins, where they can leverage the ubiquitin–proteasome system more efficiently [52].

Whilst it is likely that predictive models for selecting suitable POIs for degradation will improve as more data becomes available, the current process of PROTAC optimisation remains somewhat empirical, which generates the need for rapid synthesis and biological testing of a range of compounds.

### Section 2 – Chemical synthesis

**Figure 4:**
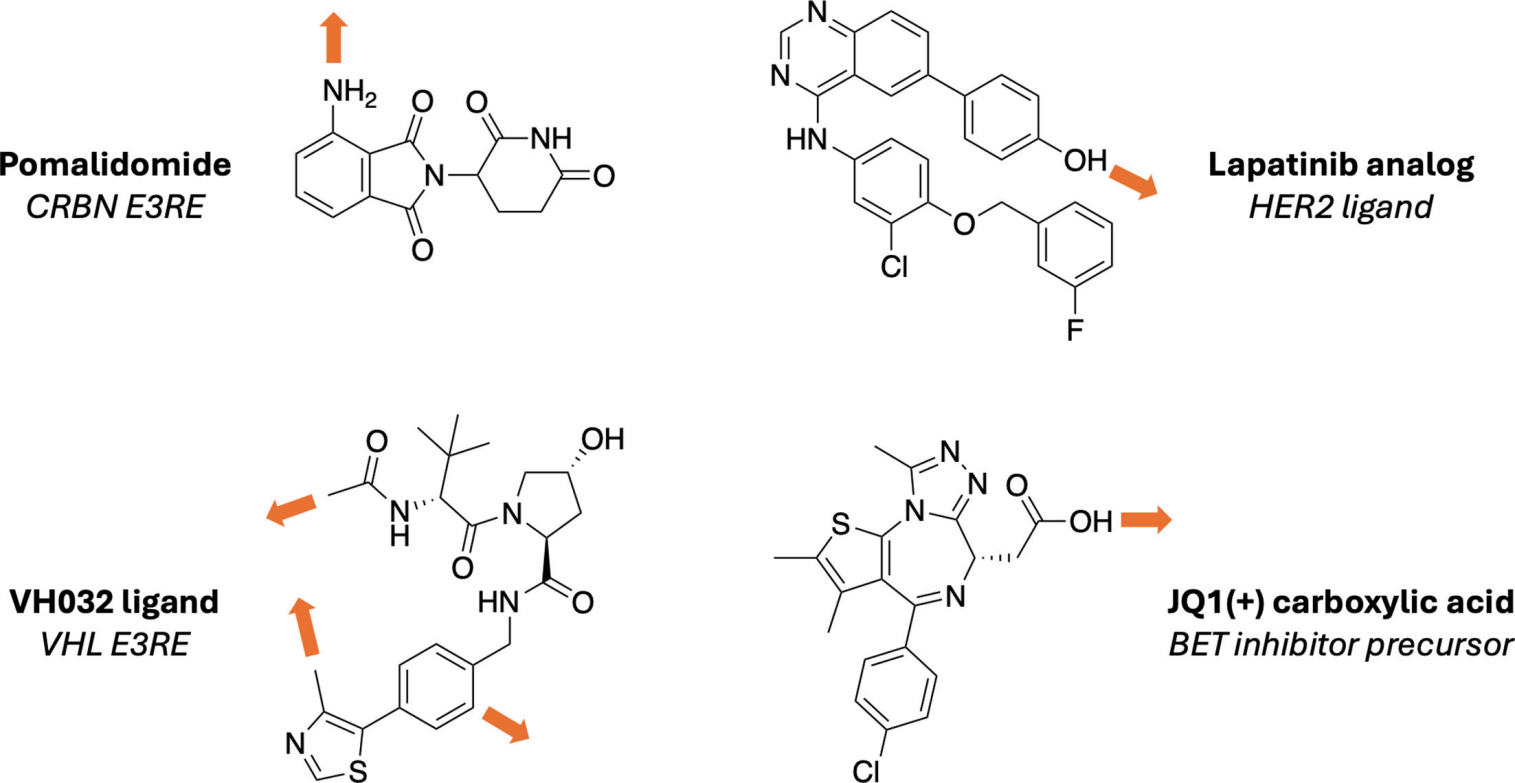
Chemical structures of E3RE and POI ligands discussed in the text. Arrows represent vectors where the ligands can be elaborated without loss of binding to their respective protein targets.

Due to the modular nature of PROTACs, their syntheses are often multi-step. A linker must be attached to both the E3RE and POI ligand without disrupting their binding to their respective targets. This involves identifying suitable linking points in each ligand which can used for elaboration (Figure 4). In addition, the linking chemistry must be suitably selective to avoid side-reactions with the array of functional groups that can be present in the two ligands. Once established, however, the modular structure of PROTACs (POI-ligand-linker-E3RE) lends itself to parallel assembly of libraries of compounds with the linker moiety as a major point of variation. In the following sections, we will highlight three different approaches that have been employed to accelerate and/or diversify the synthesis of PROTACs. First, the use of click reactions—these are selective and high-yielding transformations that are amenable to parallel chemistry and suitable for ligation of highly functionalised compounds [53]. Secondly, solid-phase organic synthesis (SPOS) allows multistep synthesis to be performed without the need for extensive purification [54]. Both parallel chemistry and SPOS have been shown to be suitable for combinatorial strategies to generate libraries of PROTAC analogues. Thirdly, DNA-encoded library (DEL) technologies, which enable the synthesis and screening of vast compound libraries, have been applied to PROTAC discovery [55,56]. In the sections below, we describe how these technologies have been applied to accelerate the synthesis and screening of PROTACs.

#### Click chemistry

**Figure 5:**
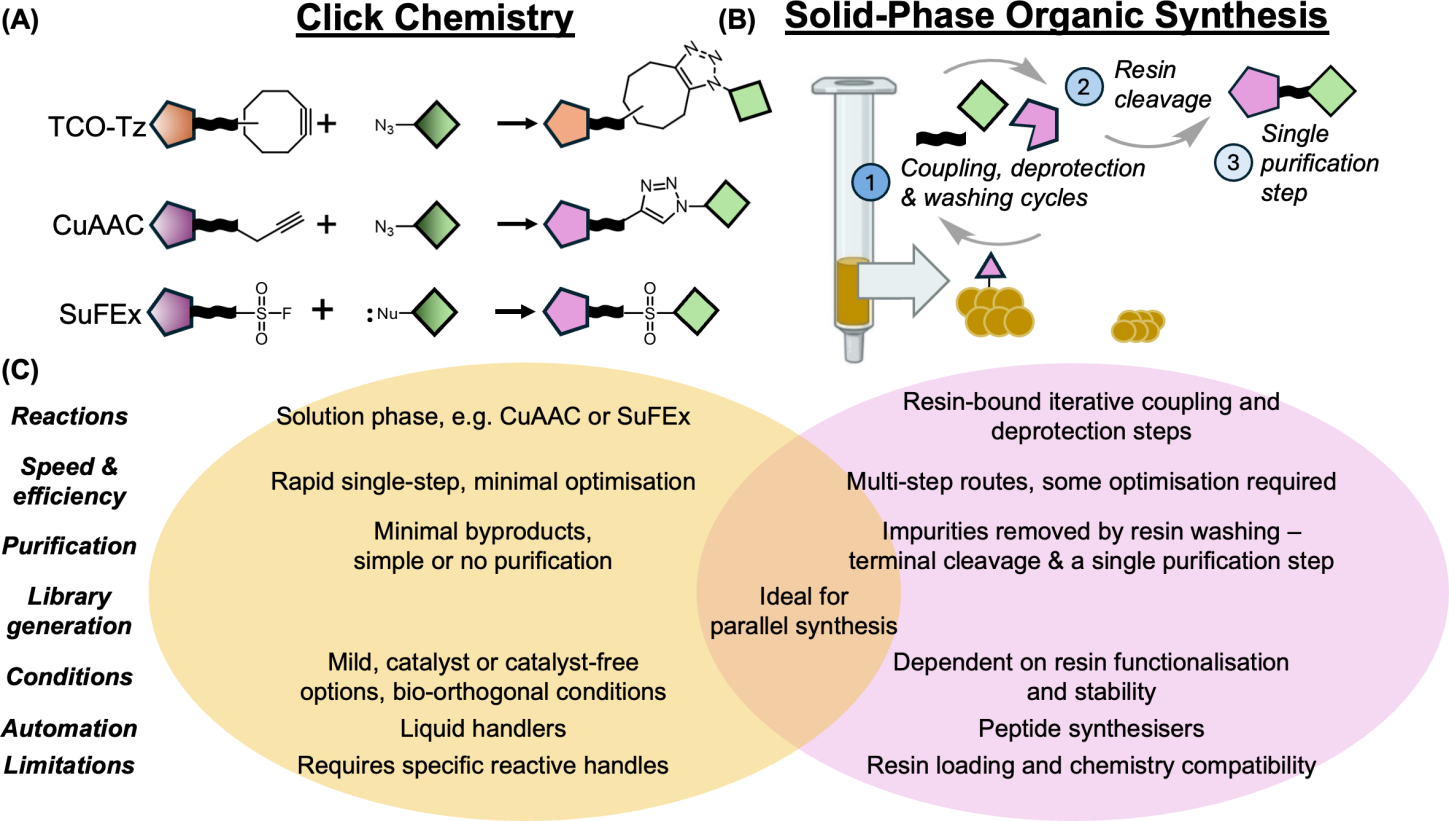
Comparison between click reactions and solid-phase organic synthesis. (**A**) Examples of some of the click chemistry reactions that have been used in PROTAC synthesis. (**B**) Schematic of the steps involved in solid phase organic synthesis. (**C**) Comparison of the two approaches.

Click chemistry refers to a series of highly selective chemical transformations. The prototypical click reaction is the copper-catalysed azido-alkyne cycloadditions [57]. However, other click reactions have been used for PROTAC synthesis, including strain-promoted azide-alkyne click chemistry [57–59], and sulfonyl fluoride exchange (Figure 5) [53,60–62].

Due to their exquisite chemoselectivity, it is possible to employ click chemistry reactions in the later stages of PROTAC synthesis. Furthermore, near-quantitative yields can be achieved under mild conditions with minimal byproducts, whereas more traditional coupling reactions, such as amide couplings, typically provide moderate yields and require additional reactants for the reaction to progress and therefore generate more byproducts. An additional benefit in comparison with amide formation is that the resulting linker does not contain an H-bond donor, and it has been suggested that this may contribute to enhanced cellular permeability [63]. Although click reactions are fast, selective, and produce relatively pure products, they do require the installation of specific reactive handles (e.g. azides), which can present their own synthetic challenges. Despite this limitation, there are many examples, summarised in recent reviews (e.g. [55]), where click chemistry has been used to synthesise analogue libraries quickly and cleanly, highlighting its utility within the PROTAC chemistry toolbox.

The selectivity of click reactions also allows them to be undertaken in the presence of biomolecules. This has even allowed click chemistry to be utilised to assemble PROTACs within cells [64]. One potential advantage of doing so is that this may provide a means to overcome the low permeability of PROTACs, since their respective starting materials are expected to have better permeability than the full PROTAC. Lebraud et al. demonstrated this concept by synthesising in-cell click-formed proteolysis targeting chimeras for BRD4 and ERK1/2 [63,65].

#### Solid-phase organic synthesis

SPOS is another well-established method where molecules can be assembled in a modular fashion on an appropriate solid support. Perhaps, the most widely used application of SPOS is in the solid-phase synthesis of peptides [56]. Throughout the synthesis, the target molecule remains bound to the solid support. This allows the synthesis to be performed in the presence of excess reagents that favour high yields. Unreacted reagents and byproducts that remain in solution can be easily removed by washing in between synthetic steps. This can accelerate the production of PROTACs, since the product can be cleaved from the solid support and purified with a single chromatographic step at the conclusion of the synthesis. The potential of SPOS in the parallel synthesis of PROTACs, where the elements are connected via amide couplings, is obvious, drawing on similarities to solid-phase peptide chemistry. However, it also allows the application of a broader arsenal of organic reactions. In this way, SPOS may offer increased diversity in library preparation [66,67]. The utility of SPOS in the assembly of PROTACs has been demonstrated using different E3REs (targeting VHL and CRBN), linkers and POI ligands (targeting BRD2 and HDAC6) [68–70]. SPOS can also be carried out in parallel to further accelerate PROTAC synthesis. In one example, a droplet microarray SPOS format was used to synthesise hundreds of PROTACs simultaneously on nanoscale, while retaining the use of characteristic intermediate washing steps to remove unreacted materials and byproducts [71]. Given the modular structure of PROTAC, SPOS affords a strategy to accelerate their production—both through parallelisation and by simplifying purification of the final products.

#### DNA-encoded library screening

A more recent innovation in the chemical synthesis of PROTACs is the use of DELs [72]. DEL synthesis has emerged as a transformative technology in traditional drug discovery, offering a highly efficient approach to identifying small-molecule binders for a wide range of targets. DELs allow for the simultaneous screening of millions or billions of compounds. DELs are synthesised using a combinatorial ‘split-and-pool’ strategy [72]. During the synthesis, each compound is covalently linked to a unique DNA sequence, which acts as a ‘barcode’. Following selection of compounds that bind to a POI, the barcode can be read using next-generation sequencing (NGS). This can then be used to decode the chemical structure of compounds that are selected from the library by binding to the target. Continuing advances in the development of ‘DNA-compatible’ reactions are broadening the scope and value of this technology [73].

**Figure 6:**
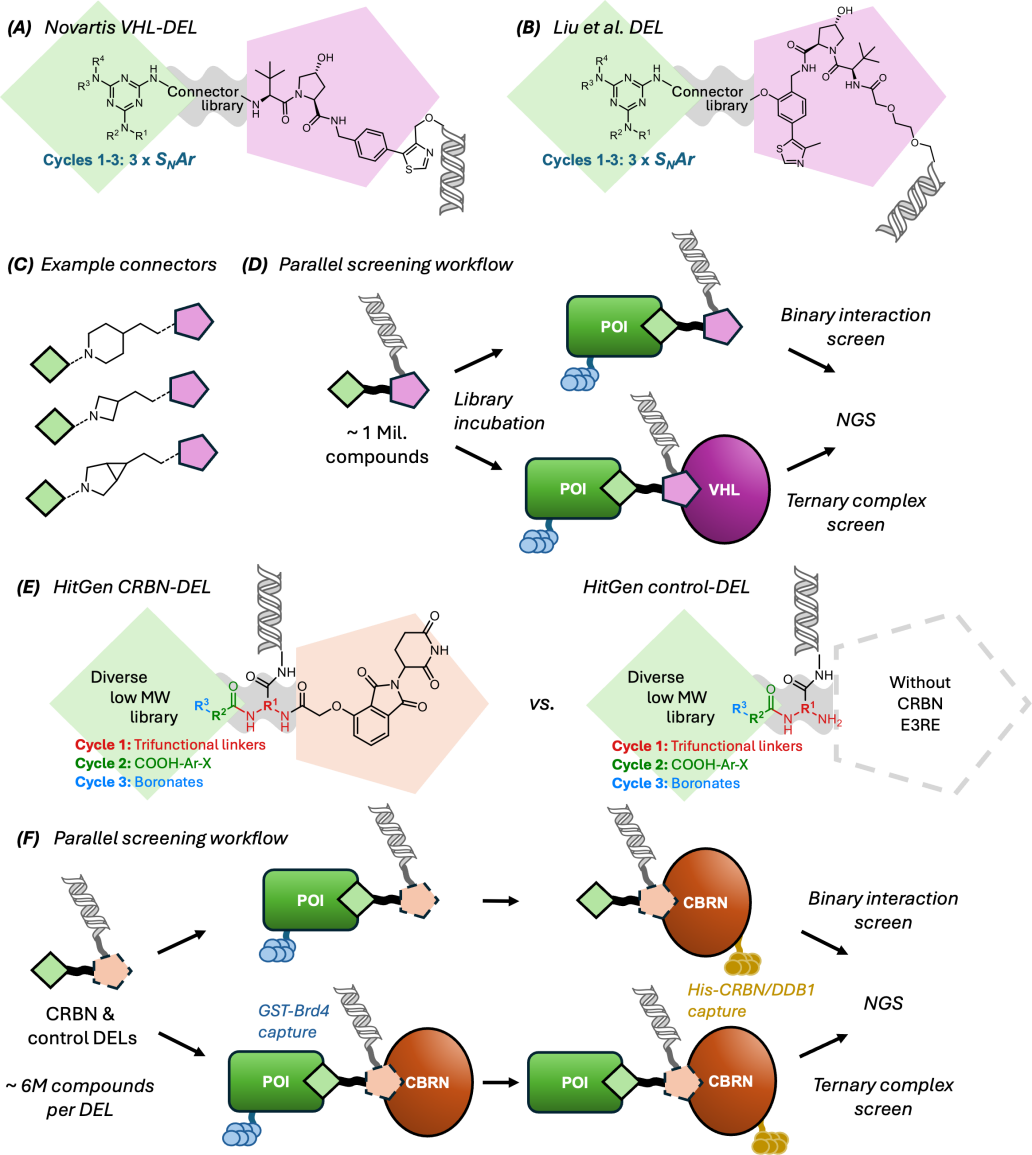
DEL workflows for PROTAC identification. (**a**) & (**b**) VHL DEL libraries showing position of DNA tag; (**c**) example connectors from both VHL-based PROTAC DELs; (**d**) VHL DEL screening cascade; (**e**) CRBN DEL and control DEL without CRBN recruiting ligand, featuring trifunctional linker with attached DNA tag; (**f**) CRBN DEL screening cascade.

This technology has recently been used for the discovery of PROTACs in a variety of ways. First, DELs can be used to identify novel ligands for either the POI or the E3 ligase. DEL hits provide an additional advantage since the hit molecules are attached via a linker to the DNA tag. This same linkage point can be used as an exit-vector in the assembly of a PROTAC [74]. DELs have also been directly used to identify ternary complex formation. This way of using DEL poses some complications, since the DNA tag must be accommodated without hindering the formation of the ternary POI-PROTAC-E3 complex. Different approaches have been described to address this challenge. In the case of the E3RE VH032, it has been shown that it is possible to modify the ligand at more than one position without disrupting binding to VHL (Figure 4). This means that two exit vectors can be functionalised simultaneously, one for linker-POI binder attachment, and the other for the DNA barcode. This strategy was used to generate VHL recruiting DELs containing about 1 million PROTAC-like members (Figure 6A–D) [55,75]. Two libraries were synthesised, which differed in where the DNA tag was attached to the VH032 structure. Novel POI binders were assembled through iterative couplings on a triazine core. A small library of ‘connectors’ was used to link the VHL E3RE to the POI binders. The DEL selection identifies compounds that induce ternary complex formation with the POI and E3 ligase but does so in the presence of the DNA barcode. A subsequent step is required to demonstrate that the compounds also bind in the absence of the DNA and are capable of inducing degradation. In these cases, DEL screening identified PROTACs that resulted in degradation of the POI used in the selection, when resynthesised off-DNA.

In contrast, E3REs that target CRBN have only a single expansion vector (Figure 4). Therefore, a different strategy is required for attaching a DNA barcode. One option is to chemically attach the DNA to the linker (Figure 6E and F). HitGen generated a CRBN-recruiting DEL in this way [59]. The DEL was constructed using three cycles of chemistry with different reagents/couplings. In addition, a control library was constructed that lacked the CRBN ligand. Four selections were carried out with both libraries against the POI (GST-tagged Brd4) and the E3 ligase (His-tagged CRBN/DDB1), to identify compounds that supported formation of a ternary complex. The resulting compounds were tested in protein degradation assays. Each of these studies was able to identify compounds that resulted in the degradation of the POI. Moreover, the PROTACs were able to degrade the POI without any further optimisation of the chemical structure.

In summary, the modular nature of PROTACs facilitates the creation of diverse compound libraries through various synthetic strategies. Click chemistry, SPOS, and DEL technology each offer unique advantages for accelerating and diversifying PROTAC synthesis. The development of novel biology methods circumventing the need for purification, a significant bottleneck in the synthetic chemistry process, has provided further opportunities to fast-track the development of PROTACs and is described below.

### Section 3 *–* Direct-to-biology

**Figure 7:**
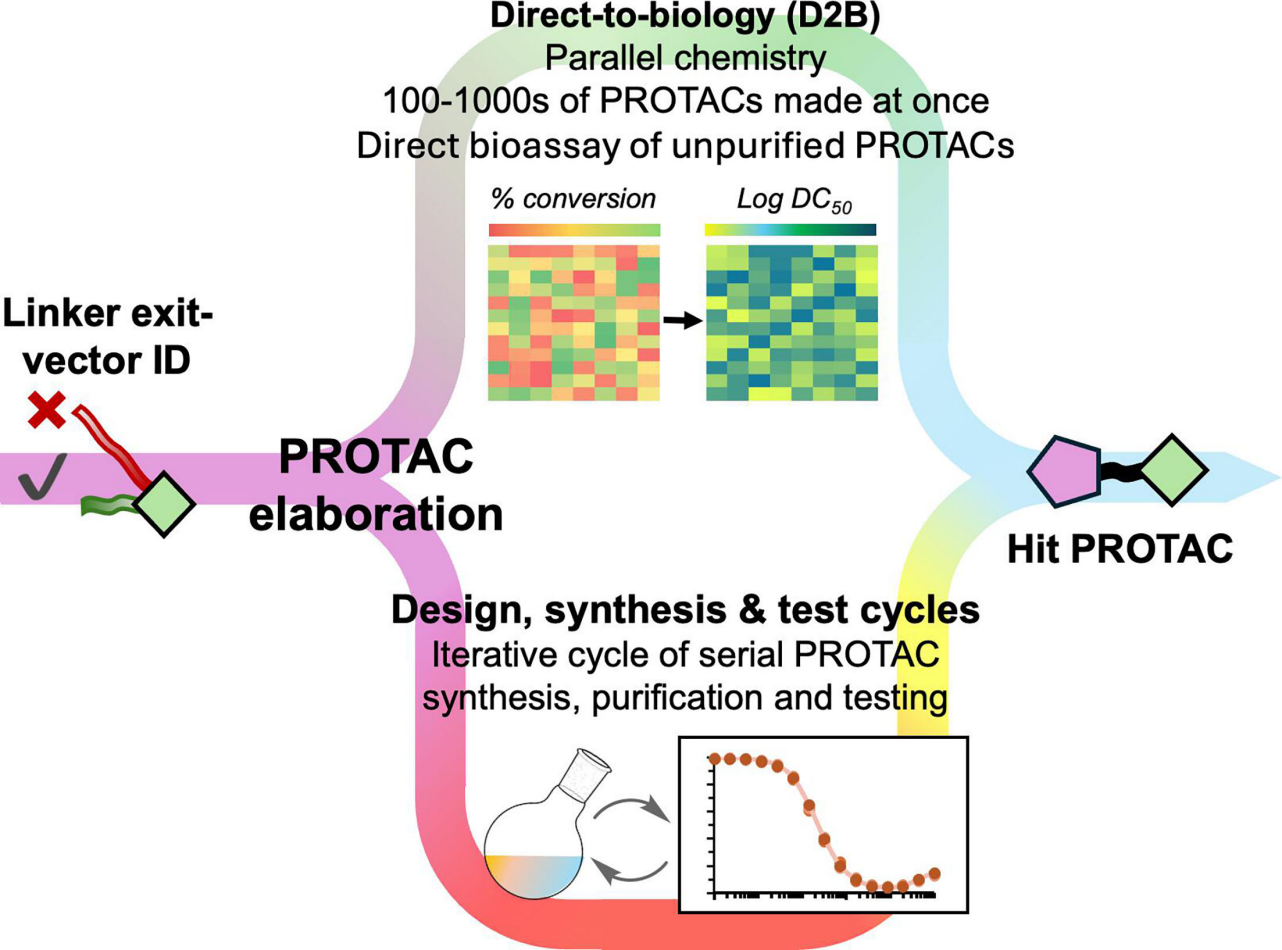
Depiction of D2B workflows vs. conventional design, synthesis and test cycles, comparing degrees of throughput.

The D2B paradigm (Figure 7) has emerged over the past decade as an approach to accelerate the screening of compound libraries [76,77]. A common implementation of D2B involves screening of crude reaction mixtures (CRM). These CRMs represent individual reactions, which are often synthesised in parallel as part of a library. Since screening techniques typically requires a relatively small amount of material, the synthesis can be performed on a small scale (typically 1 μmol or less). An early example of this type of approach is off-rate screening (ORS) by surface plasmon resonance [78]. Numerous other D2B methods have now been reported [79,80]. These include fluorescence antibody-based methods such as Amplified Luminescent Proximity Homogenous Assay Screen (AlphaScreen™), time-resolved fluorescence energy transfer, and NanoLuciferase based assays such as nano bioluminescence resonance energy transfer (NanoBRET) and Nano-Glo HiBit technology [81,82]. These methods are generally suitable for automation and use in high-throughput formats and a variety of different approaches have been used in the D2B examples described below.

**Figure 8:**
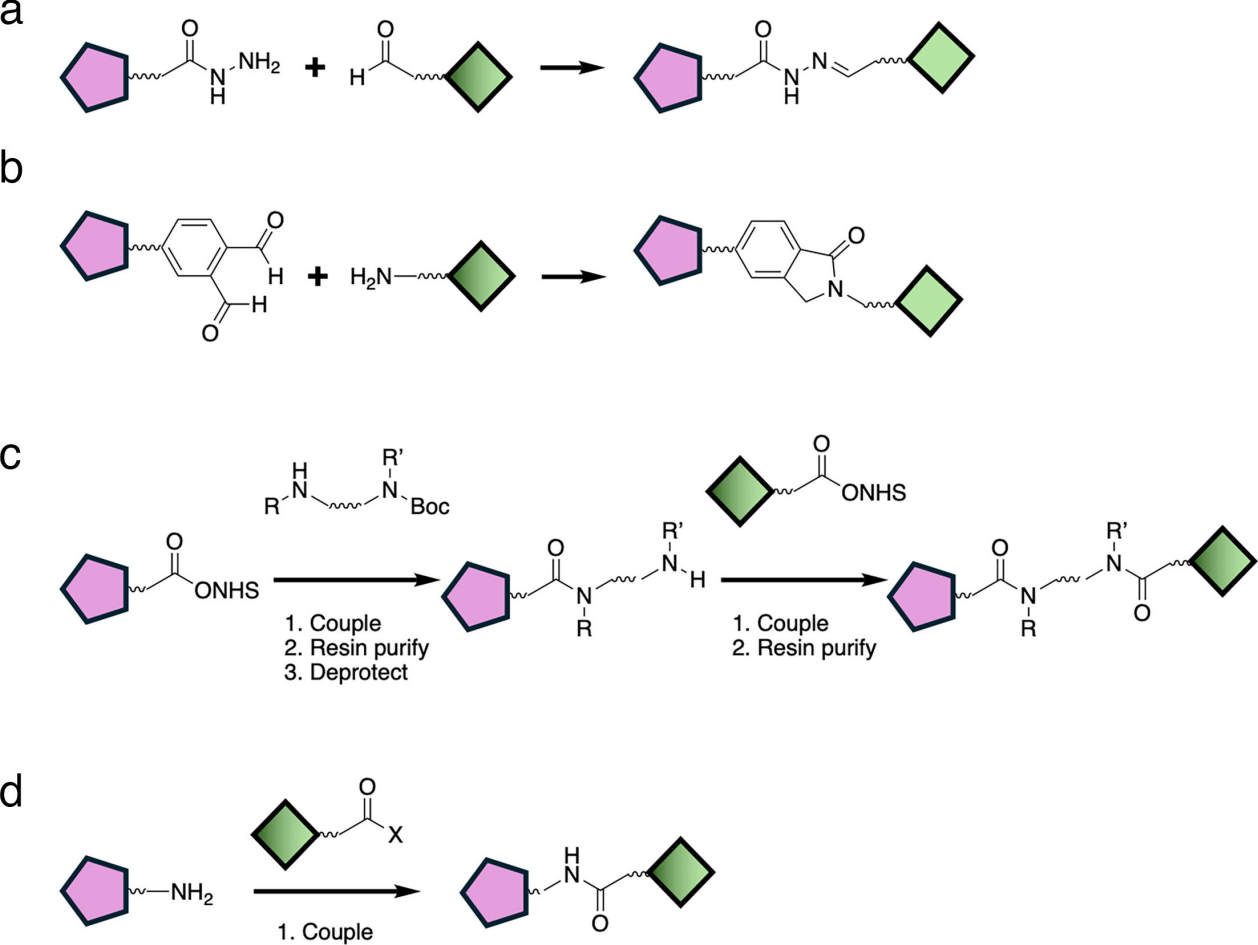
Chemical synthesis of D2B libraries. (**a**) RAPID-Tac acyl hydrazone synthesis. (**b**) Second-generation RAPID-Tac phthalimidine synthesis. (**c**) Telescoped three-step amide synthesis employed by Janssen. (**d**) One-step amide synthesis employed by GSK and AstraZeneca.

One of the first examples of using the D2B approach to accelerate PROTAC discovery was reported by Tang and co-workers (Figure 8A) [83]. In this workflow, which they termed Rapid-TAC, a POI ligand bearing a hydrazide was reacted with a library of E3 ligase–linker conjugates having a terminal aldehyde to generate a library of acyl hydrazones. The advantage of this strategy is that the reactions were found to show high conversion to the desired product with minimal byproducts. The resulting CRMs were suitable for testing in a cell-based assay. This approach was trialled using the estrogen receptor as the POI. A library of almost 100 different PROTACs was prepared using a small library of known ER ligands and a library of E3REs with a linker attached. This approach was successful in identifying an effective PROTAC (DC_50_~10 nM; D_max_ > 95%). A drawback of this method is the fact that the acyl hydrazone linker is labile, and therefore a second round of synthesis was required find a more suitable and stable linker than the acyl hydrazone. In this example, the acyl hydrazone could be replaced by an amide without loss of activity. To circumvent the requirement for this second step, the same group later reported the use of a different chemistry (Figure 8B). The revised Rapid-TAC approach employed *ortho*-phthalaldehyde on the POI ligand and an amine on the E3RE-linker to generate a phthalimidine [84,85]. This chemistry afforded similarly high yields as the original Rapid-TAC approach, minimal side products, and the resultant CRMs were suitable for testing in cellular assays. Using this approach, the authors were able to generate active PROTACs that were able to degrade two different POI targets, namely the androgen receptor and BRD4.

Four separate, but related, approaches were reported by Yan et al. and three pharmaceutical companies, Janssen, GSK, and AstraZeneca (Figure 8C and D) [86–89]. They shared a similar premise in that libraries of compounds were synthesised in parallel and tested without purification using a D2B approach. Each used a modular synthesis to generate libraries of PROTACs targeting well-characterised POI targets and E3REs targeting either VHL or CRBN. In each case, amide-couplings were used to assemble the PROTACs. Subsequently, a range of biological assays was used to investigate target engagement, degradation, and cell toxicity.

In the Janssen study, a three-step synthesis was used [87]. Reaction conditions for amide coupling were first optimised. These were used for a library of 91 diamine linkers, which were amide-coupled to an E3RE and then to a POI ligand. Two CRBN ligands, namely tolyl dihydrouracil and pomalidomide, were used as the E3REs and JQ1 was used as the POI target to generate 182 PROTACs that target BRD4. A resin-based purification was used after each amide coupling, but no chromatographic purification was required. Reaction conversion across the telescoped reaction sequence was ~30% as determined using LCMS, and the desired products were identified in ~80% of reactions. The PROTACs were tested without further purification in four separate assays. E3 engagement was assessed in a NanoBRET assay in live and permeabilised cells, providing a means to assess PROTAC permeability. Degradation was determined using a BRD4 HiBiT assay, and toxicity was measured using a Cell Titre Glo (CTG) assay. A good correlation was observed between the readouts of CRMs and purified compounds in both the target engagement and degradation assays, whilst degradation was not correlated with general cell toxicity. Overall, the D_max_ calculated for purified compounds was found to be higher than that for CRMs, which was attributed to the presence of impurities in the CRMs [87]. The other two studies employed libraries of E3RE-linker conjugates in the synthesis, which were coupled to a POI ligand using amide chemistry. This meant that the libraries could be generated in a single step. This resulted in higher conversion (≥80%) than was observed for the telescoped three step synthesis. Again, the CRMs were tested without any further purification. CRMs were analysed for degradation and cellular toxicity using HiBit and CTG assays, respectively.

In the GSK study, human epidermal growth factor receptor 2 was first chosen as the model system with lapatinib bearing a PEG-carboxylic acid reactive handle as the POI ligand [88]. Chemistry was designed to be performed in 1536-well plates in volumes of 5 μL, which allowed synthesis on 150 nmol scale. First, different amide coupling conditions were evaluated to find the conditions showing the least general cellular toxicity. These were used to synthesise a small library of PROTACs, by coupling the lapatinib-acid with 29 E3RE-linker amines. Analysis of the CRMs revealed that the positive control PROTAC was identified as a degrader from the library. In addition, several novel PROTACs were identified. In this case, a more pronounced Hook effect was observed in analysis of the CRMs compared with the pure compounds, which was again attributed to the presence of impurities.

The identification of novel BRD4 PROTACs followed the same approach. A 186-membered library was prepared by reacting 87 E3-linker amines with the BRD4 ligand iBET469, which was functionalised at three different exit vectors. Again, this led to the identification of novel BRD4 PROTACs. Due to the miniaturisation, a full library of PROTACs could be synthesised with as little as 10 mg of each POI-acid intermediate. In total, over 600 compounds were synthesised and tested, accelerating the workflow by >100-fold in comparison with synthesis of each compound individually. The AstraZeneca study used BRD4 as the model system [89]. Two different JQ1-acids were used as the POI ligands and reacted with 34 E3RE-linker amines on a ~110-nmol scale. Again, several PROTACs were identified that showed good D_max_ and DC_50_. Resynthesis on a larger scale (20 μmol) confirmed that the potencies derived from the analysis of the CRMs were reproducible across a range of D_max_/DC_50_ values, although D_max_ tended to be lower in the CRM analysis. Moreover, by assessing degradation in mixtures that were made by diluting a purified PROTAC with a mixture of reagents simulating the CRM, it was determined that even with conversions as low as 10%, degradation could still be reliably observed.

More recently, Yan et al. reported a modular three-step synthesis that utilises a light-induced photoclick amide-coupling reaction between primary amines and *O*-nitrobenzyl alcohol-derived *N*-hydroxysuccinimide esters [86]. Again, this approach resulted in high yields of the desired products with minimal byproduct impurities. Following library chemistry, potent and selective degraders of CDK9 were identified.

A key assumption in many of these methods is that the readout from the assay will be dominated by the compound in the CRM with the highest affinity/potency for the target. For conventional small molecules, whose activity is driven by occupancy, it has been demonstrated that this assumption is reasonable. However, for PROTACs, with their event-driven pharmacology, the situation is more complex. To assess the efficacy of a PROTAC, it is necessary to measure POI degradation in cellular assays. This means that cellular toxicity, which may result either from the PROTAC itself or another component of the CRM, must be considered. Cellular permeability is also a significant factor in PROTAC efficacy, which is not the case for D2B methods based on biophysical binding assays such as ORS. Moreover, starting materials may have better cell permeability than the larger PROTAC, while binding to either the POI or the E3 ligase with similar affinity. Therefore, CRMs from PROTAC synthesis can cause significant interference in assays. For instance, if there were unreacted ligands present that compete with the PROTAC for binding to the POI or E3 ligase, then this could inhibit ternary complex formation. However, since the goal of the D2B approaches is to accelerate the identification of functional degraders, CRMs that do not give a positive readout are typically not pursued, and the rate of false-negative readouts is generally not characterised. Nonetheless, each of the studies above demonstrated the feasibility of applying D2B approaches in identifying functional PROTACs. The D2B methodology significantly accelerates the rate at which PROTACs can be synthesised and evaluated. Moreover, it provides a wealth of data on the effects of compound properties and linker composition on degradation. Such data offer the opportunity to move beyond the current empirical strategies for PROTAC optimisation and inform more systematic predictive models, which may further accelerate PROTAC development.

## Conclusion

PROTACs have emerged as a ground-breaking modality in chemical biology and drug discovery. These bifunctional molecules work by hijacking the ubiquitin proteasome system to degrade target proteins, offering a novel approach to treating diseases. While their clinical success remains uncertain, PROTACs have now entered the clinic, suggesting that the first approved PROTAC therapeutic drug may be imminent, which would mark a significant milestone in this field.

Accelerating PROTAC development is crucial for several reasons. First, by accelerating the discovery phase, the cost is reduced. Currently, the cost of bringing new drugs to market averages >$2 billion [90]. Additionally, reducing both resources and the environmental impact of drug discovery programmes are important [91,92]. Moreover, the methods described above can generate data that link to artificial intelligence and machine-learning-driven methods, which may enhance the precision of target identification and lead optimisation [93]. Given the unique mechanism of action of PROTACs, their potential to reduce drug dosages, and their ability to target proteins previously deemed ‘undruggable’, accelerating their development is crucial. A streamlined pipeline can improve patient access to life-saving medications, addressing urgent health needs more promptly. Hence, rapid advancement is essential to fully explore and realise the clinical benefits that PROTACs can offer.

This review focusses predominantly on PROTACs that recruit two E3 ligases—VHL and CRBN. However, many of the methods that have been used to accelerate PROTAC development are modular, and it is likely that they can be translated to other E3 ligases. Expanding the arsenal of E3REs to access different E3 ligases will not only provide new opportunities but also present new challenges. Considering the vast differences in structure, modes of regulation, physiological roles, and tissue-specific expression of E3 ligases, understanding the cellular localisation and expression levels of both the E3 and the target POI might enable the development of tissue-specific PROTACs with increased drug efficacy and fewer side effects. This may also assist in circumventing some of the challenges presented by mutations in E3 ligases, which can significantly impact the binding and efficacy of PROTACs, leading to resistance [94,95]. For instance, preclinical studies have shown the development of resistance to VHL- and CRBN-based PROTACs, due to mutations and reduced expression observed in the respective E3 ligases or the POI. Early identification and understanding of these mutations allow researchers to address these problems, e.g. by designing PROTACs that target less mutation-prone sites of POIs to prevent the emergence of resistance.

In this review, we have highlighted progress in three critical areas to accelerate PROTAC development. A rigorous target selection process ensures that a drug discovery programme is on the right track from the beginning. The appropriate selection of both the POI and E3 ligase is key to the development of efficacious and safe drugs with minimal side effects. Another constraint in traditional PROTAC drug discovery programmes are the time and resources required for synthesis, purification, and testing for each individual molecule and time wasted in pharmacological dead-ends. High-throughput screening methods, as well as automated synthesis and testing platforms, have been reported to overcome these issues, allowing for the rapid evaluation of numerous PROTAC candidates. These approaches will accelerate the development of PROTACs but are also likely to be readily transferable to other heterobivalent modalities that simultaneously engage two distinct biological targets. For instance, other degrader modalities such as molecular glues, specific and non-genetic inhibitor of apoptosis protein-dependent protein erasers (SNIPERs), LYTACs, and autophagy-targeting chimeras (AUTACs) can leverage these advancements to streamline development. Overall, these approaches allow researchers to rapidly assess the efficacy and safety profiles of numerous heterobivalent ligands, facilitating the identification of the most promising candidates for further development. Collectively, these advancements will drive the field of PROTACs forward, enabling the rapid development of more effective and targeted therapies for a range of diseases.

## Abbreviations

BET: Bromodomain and Extra Terminal
BRD4: Bromodomain-containing protein 4
CRBN: cereblon
CRM: crude reaction mixture
CTG: Cell Titre Glo
D2B: direct-to-biology
DEL: DNA-encoded library
EGFR: epidermal growth factor receptor
E3RE: E3 recruiting element
GPCRs: G protein-coupled receptor
LYTAC: lysosome-targeting chimera
MP: mutant protein
NGS: next generation sequencing
NanoBRET: nano bioluminescence resonance energy transfer
ORS: off-rate screening
POI: protein of interest
PROTACs: Proteolysis targeting chimeras
RTKs: receptor tyrosine kinases
SPOS: solid-phase organic synthesis
TPD: targeted protein degradation
VHL: von Hippel-Lindau
tTPD: tag-targeted protein degraders
